# Polycystin-2 mediates mechanical tension-induced osteogenic differentiation of human adipose-derived stem cells by activating transcriptional co-activator with PDZ-binding motif

**DOI:** 10.3389/fphys.2022.917510

**Published:** 2022-08-24

**Authors:** Liang Wang, Yahui Lu, Guanhui Cai, Hongyu Chen, Gen Li, Luwei Liu, Lian Sun, Zhaolan Guan, Wen Sun, Chunyang Zhao, Hua Wang

**Affiliations:** ^1^ Jiangsu Key Laboratory of Oral Diseases, Jiangsu Province Engineering Research Center of Stomatological Translational Medicine, Department of Orthodontics, Affiliated Hospital of Stomatology, Nanjing Medical University, Nanjing, China; ^2^ Department of Stomatology, Affiliated Hospital of Nantong University, Nantong, China

**Keywords:** mechanical stimuli, polycystin-2, human adipose-derived stem cells, osteogenesis, TAZ (transcriptional co-activator with PDZ-binding motif)

## Abstract

Human adipose-derived stem cells (hASCs) have multi-directional differentiation potential including osteogenic differentiation. Mechanical stimulation is thought to be a key regulator of bone remodeling and has been proved to promote osteogenic differentiation of mesenchymal stem cells. However, the mechanism how mechanical tension-induced osteogenesis of hASCs still remains poor understood. Polycystin-2 (PC2), a member of the transient receptor potential polycystic (TRPP) family, is involved in cilia-mediated mechanical transduction. To understand the role of PC2 in osteogenic differentiation under mechanical stimuli in hASCs, PKD2 gene was stably silenced by using lentivirus-mediated shRNA technology. The results showed that mechanical tension sufficiently enhanced osteogenic differentiation but hardly affected proliferation of hASCs. Silencing PKD2 gene caused hASCs to lose the ability of sensing mechanical stimuli and subsequently promoting osteogenesis. PC2 knock-out also reduced the cilia population frequency and cilia length in hASCs. TAZ (transcriptional coactivator with PDZ-binding motif, also known as Wwtr1) could mediate the genes regulation and biological functions of mechanotransduction signal pathway. Here, mechanical tension also enhanced TAZ nuclear translocation of hASCs. PC2 knock-out blocked tension-induced upregulation of nuclear TAZ and suppress tension-induced osteogenesis. TAZ could directly interact with Runx2, and inhibiting TAZ could suppress tension-induced upregulation of Runx2 expression. In summary, our findings demonstrated that PC2 mediate mechanical tension-induced osteogenic differentiation of hASCs by activating TAZ.

## Highlights

- Mechanical tension could enhance osteogenic differentiation of human adipose-derived stem cells (hASCs).
- Polycystin-2 (PC2) was involved in cilia-mediated mechanical transduction and PC2 knock-out blocked tension-induced upregulation of osteogenesis.
- PC2 mediated tension-induced osteogenic differentiation of hASCs via upregulating nuclear TAZ expression.

## Introduction

Craniofacial defects caused by maxillofacial tumors, infections etc often cause soft tissue or bone deficits, or a combination of both, which affects the physical health of patients (Zhu et al., 2018; Kim et al., 2021; Kim et al., 2022). The reconstruction of bone defects in this region presents many challenges due to the unique anatomy and the presence of a vital structure (Nyberg et al., 2016; Oh, 2018). Recently, repairing bone defects by culturing biologically active tissues *in vitro* is becoming a research hotspot (Lin et al., 2020). Mesenchymal stem cells (MSCs) are most used in bone tissue engineering combining with biomaterial scaffolds to reshape bone tissue morphology (Seitz et al., 2005; Wang et al., 2015). Human adipose-derived stem cells (hASCs) have multi-directional differentiation including osteogenic differentiation, so hASCs constitute a major field of research in seed cells of bone tissue engineering. In addition, hASCs have abundant sources which can be isolated extremely conveniently and widely. Therefore, it is of great significance to study the development trend of bone defects in hASCs(Chen et al., 2017; Gentile et al., 2019).

The differentiation of stem cells can be affected by the microenvironment in which the cells are located through a combination of factors which are biochemical, biophysical and biomechanical (Thompson et al., 2012; Marrelli et al., 2018; Fang et al., 2019). Numerous scholars discovered that mechanical stimuli could change the morphology, and structure cells (Wang et al., 2014; Vassaux and Milan, 2017). It is reported that mechanical stimulation is also an important epigenetic factor regulating stem cell differentiation (Wu et al., 2018). Several studies have shown that ASCs undergo increased osteogenic differentiation under mechanical strain, and the process involves the complex morphology of the cytoskeleton and biochemical changes (Fang et al., 2019; Luo et al., 2020).

Polycystin-2 (PC2) is a transmembrane protein encoded by the PKD2 gene expressed in a variety of cells including ASCs(Hasan et al., 2019). In epithelial cells containing primary cilia, PC2 is colocalized with ciliary polycystin-1 and functions as a component of a mechanosensory complex (Thompson et al., 2021). Conditionally inactivated PKD2 in mature osteoblasts resulted in bone loss and impaired biomechanical properties of bone (Xiao et al., 2014). PC2 is involved in mediating the release of calcium from intracellular storage and may contribute to the transmission of mechanical stimuli through primary cilia (Retailleau and Duprat, 2014; Thompson et al., 2021). Based on previous results, we believe that PC2 may play an important role in regulating the differentiation of ASCs under mechanical stimuli.

Mechanical modulation of cells involves complex biomechanical signaling transduction. TAZ (transcriptional coactivator with PDZ-binding motif), a key downstream effector of the Hippo pathway, mediates the major genes regulation and biological functions of mechanotransduction signal pathway (Li et al., 2020). It is reported that TAZ could bind to PC1 C-terminal tail (PC1-CTT) to promote nuclear translocation, and bind to PC2-CTT to enhance PC2 degradation, which can stimulate osteoblastogenesis (Tian et al., 2007; Merrick et al., 2019). Therefore, PC2 and TAZ may be an essential role in mechanical-induced osteogenic differentiation. However, research in this field is still incomplete due to the diversity of cells and additional studies should be performed.

Based on these previous results, we hypothesized that PC2 regulates mechanical tension-induced osteogenic differentiation of hASCs and this process is related to TAZ. Here, we isolated human adipose-derived stem cell and identified its capable of osteogenic differentiation under mechanical stimulation. Silencing PKD2 gene by lentivirus-mediated shRNA impaired the effect of mechanical tension-induced osteogenic differentiation and TAZ nuclear relocation in hASCs. We further revealed that mechanical tension-induced osteogenic differentiation could be also reduced by silencing PKD2 gene. The results have guiding significance for the osteogenic differentiation of hASCs under the mechanical tension in the bone regeneration.

## Material and method

### Cell culture

Fat tissue was obtained from six donors who underwent flap reconstruction in the Oral and Maxillofacial Surgery Department at the Affiliated Stomatological Hospital of Nanjing Medical University. This study was approved by the ethics committee of Nanjing Medical University, and all patients signed informed consent. Adipose tissue was isolated from flaps and hASCS were harvested as reported by Luo et al. (Luo et al., 2019). Cells were cultured in alpha-modified Eagle’s medium (α- MEM, Gibco BRL, United States ) containing 10% fetal bovine serum (FBS; Gibco BRL, United States ) and 1% penicillin and streptomycin (Hyclone) at 37°C in a 5% CO_2_ incubator. In this study, 3-4 passage cells were used in the following experiments.

### Multilineage potential of human adipose-derived stem cells

hASCs were induced toward adipogenic and osteogenic to confirm their multilineage potential. Adipogenic medium (10% FBS, 1 μM dexamethasone, 200 μM indomethacin, 10 mg/L insulin, and 0.5 mM 3-isobutyl-1-methylxanthine in α-MEM) was used to induce adipogenic differentiation of hASCs. After 14-days’ culture, hASCs were stained with Oil Red O to determine the formation of lipid droplets. Osteogenic medium (10% FBS, 0.1 μM dexamethasone, 10 mM β-glycerol phosphate, and 50 μM vitamin C in α-MEM) was used to induce osteogenic differentiation of hASCs. After 21-days’ culture, hASCs were stained with Alizarin Red to confirm the existence of mineralized nodules.

### Cyclic strain loading on human adipose-derived stem cells

hASCs were seeded on silicone rubber BioFlex^®^ Culture Plates (Flexcell, United States ) at a density of 5×10^4^ cells per well for proliferation studies and 1 × 10^5^ cells per well for signal transduction studies. Cells were incubated in general growth medium. Cyclic sinusoidal continuous tensile strain was applied (10%, 0.5 Hz, 4h/day) with Flexcell^®^ FX- 5000™ Tension System (Flexcell International Corporation, United States ). The control groups were cultured in α-MEM supplemented with 10% FBS without cyclic strain loading.

### Cell counting kit-8

After being subjected to mechanical tension for 4 h each day, hASCs were incubated with Cell Counting Kit-8 (CCK8) solution (Dojindo, Japan) at 37°C for 2 h. The optical density (OD) value was tested by a microplate reader at 450 nm.

### Surviving cell counting

The surviving cells in each well were manually counted under microscopy. 10 μl cell suspension and 10 μl 0.4% trypan blue staining solution were taken out and dropped into the cell counting plate. Observed under the microscope, those with strong refractive index and no staining are living cells, and those with blue staining are dead cells.

### Real-time polymerase chain reaction

Total RNA was isolated from hASCs by TRIzol Reagent (Invitrogen). and detected by a NanoVue™ Plus spectrophotometer (GE Healthcare Life Sciences, United States ) to determine purity at 260/280 nm. Complementary DNA (cDNA) was synthesized from RNA using the TaKaRa Primescript RT Master Mix Kit (catalog number: RR036A; TaKaRa). Relative transcript levels were measured by quantitative polymerase chain reaction (PCR) using ABI PRISM 7300sequence detection system (Applied Biosystems, Foster City, CA, United States ), according to the manufacturer’s protocol for SYBR-Green (Roche). The messenger RNA (mRNA) expression levels of Runx2, Osteocalcin (OCN), Polycystin-2 were normalized with glyceraldehyde 3-phosphate dehydrogenase (GAPDH). Sense and anti-sense primers (Invitrogen) used in the study were listed as follows:

Runx2: 5′-TTC​ACC​TTG​ACC​ATA​ACC​GTC-3′ and 5′-GGC​GGT​CAG​AGA​ACA​AAC​TAG-3′;
Osteocalcin: 5′-CAC​TCC​TCG​CCC​TAT​TGG​C-3′, and 5′-CCC​TCC​TGC​TTG​GAC​ACA​AAG-3′;
Polycystin-2: 5′-AGC​GAG​CCA​AAC​TGA​AGA​G-3′, and 5′-ATT​CCC​AGC​GTT​CCA​ACT​C-3′;
GAPDH: 5′-GAA​CGG​GAA​GCT​CAC​TGG-3′ and 5′-GCC​TGC​TTC​ACC​ACC​TTC​T-3′.

### Western blot

hASCs proteins were separated by SDS–PAGE and immunoblotting was carried out as described previously (Wang et al., 2014). Briefly, the membranes were incubated overnight with the primary antibodies: anti-polycystin-2 (28331; SANT CRUZ), anti-TAZ (4883; CST), anti-H3 (4499; CST), and p-TAZ (59971; CST). The membranes were washed with Tris-buffered saline containing 0.05% Tween 20. Appropriate secondary antibody was used to incubate the blots at room temperature for 1 h. Protein bands were visualized by Millipore Immobilon ECL (WBKLS0100) in Tanon 5200 chemiluminescence imaging system.

### Immunofluorescence

hASCs were seeded on coverslips in 12-well plates to detect the effect of osteoinduction on polycystin-2 and the effect of PC2 knockdown on primary cilia formation by immunofluorescence. After 48 h, hASCs were fixed with 4% paraformal dehyde for 15 min and washed three times with PBS. Triton X-100 (0.5%, Sigma-Aldrich, United States ) was added to each well for 20 min at room temperature. After washing three times with PBS, hASCs were blocked with goat serum for 1 h. Primary antibodies specific for polycystin-2 (1:50, SANTA CRUZ, United States ) and acetylated α-tubulin antibody (diluted 1:100) (Sigma, Catalog #T7451) were added to the cells and then incubated overnight at 4°C. After washing three times with PBS Tween-20, fluorescent Cy3 secondary antibodies (1:50, Proteintech, United States ) were added and incubated for 1 h at 37 °C in the dark. The nucleus was then restained with DAPI (Sigma-Aldrich, United States ). Cells were subsequently viewed by fluorescence microscopy (ZEISS, Oberkochen, Germany).

### Lentivirus transfection

The lentiviral vector containing short hairpin (sh)RNAs against human polycystin-2 and TAZ gene were provided by GeneChem (Shanghai, China). The target shRNAs were designed as follows:

Pkd2-shRNA: 5′- TTT​GAT​TTC​TTC​CTG​GCA​GCC​TGT​GAG​AT-3′;
TAZ-shRNA: 5′ - AGG​TAC​TTC​CTC​AAT​CAC​A-3′;

Con-shRNA: 5′-TTC​TCC​GAA​CGT​GTC​ACG​T-3′. Lentiviral frame plasmid GV493 (hU6-MCS-CBh-gcGFP- IRES-puromycin) was used to generate polycystin-2 shRNA, TAZ shRNA and control shRNA expression vector. All lentiviruses containing a GFP gene sequence for better observation. hASCs were transfected with polycystin-2 or TAZ lentiviruses for 10 h after 30%–40% confluence.

### Alkaline phosphatase staining and alkaline phosphatase activity assay

Tension group and no-tension group cells were washed by ice-cold PBS twice and fixed in 1 ml 4% paraformaldehyde for 20 min at room temperature. Alkaline phosphatase (ALP) staining was performed using BCIP/NBT Alkaline Phosphatase Color Development Kit (C3206; Beyotime, Shanghai, China) according to the manufacturer’s protocol. Then, photos were taken by a scanner (GE Image scanner III). Cellular ALP activity was detected using Alkaline Phosphatase Assay Kit (A509-2; Jian-Cheng Bioengineering Institute, Nanjing, China) and the procedure was described before (Li et al., 2020). Finally, the absorbance values related to ALP activity were recorded at 520 nm with the microtiter plate spectrophotometer (Spectrama).

### Dual-luciferase reporter assay

The promoter Runx2 overexpression and transcription factor TAZ overexpression plasmid were constructed. 293T cells seeded in 24-well plates were transfected with 6OSE2-luciferase reporter, Runx2, and TAZ expression vectors using the X-tremegene HP Reagent (Roche). Cells were lysed 24 h after transfection and the Dual Luciferase Reporter Assay System (Promega) were used to assess the Renilla and Firefly luciferase activity.

### Statistical analysis

The data were presented as mean ± SEM and analyzed using SPSS software or GraphPad Prism 5. We analyzed the statistically significant differences using Student’s t test or analysis of variance. *p < 0.05 was considered to indicate significant statistical difference for all experiments.

## Results

### Polycystin-2 expression was related to the osteogenic differentiation of human adipose-derived stem cells

**FIGURE 1:**
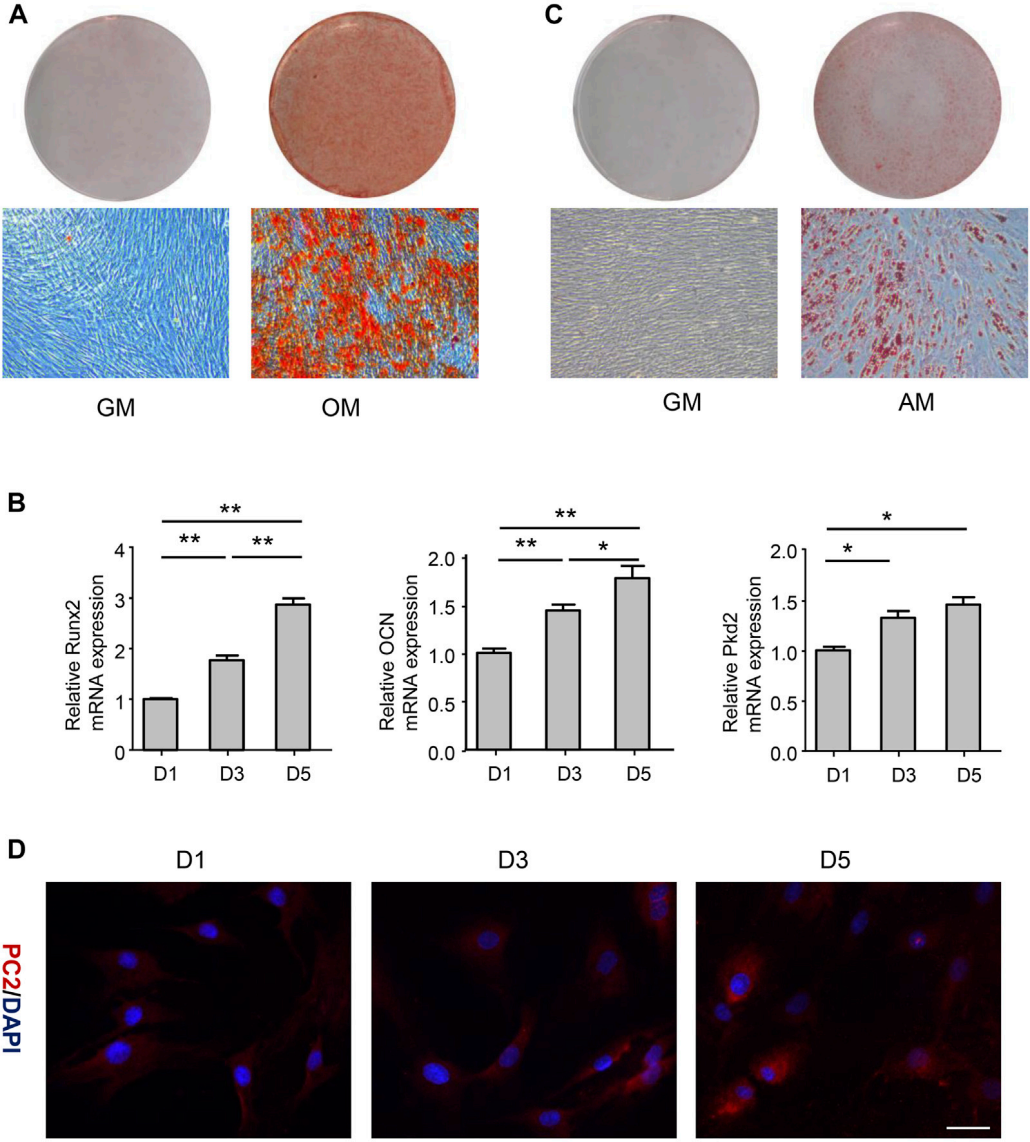
PC2 expression was related to the osteogenic differentiation of hASCs. **(A)** hASCs were cultured in the osteogenic medium (OM) for 21 days, and Alizarin Red S staining was performed. **(B)** The mRNA levels of Runx2, OCN and PKD2 were detected by real-time polymerase chain reaction (PCR) after culturing hASCs in OM for 1-, 3-, or 5-day. **(C)** hASCs were cultured in the adipogenic medium (AM) for 14 days, and Oil Red O staining was performed **(D)** Immunofluorescence staining of PC2 was detected after culturing hASCs in OM for 1-, 3-, or 5-day. Scale bar: 200 μm. Values are the mean ± SEM. *p < 0.05, **p < 0.01. Experiments = 3.

To investigate whether hASCs could differentiate into multiple cell lineages, hASCs were cultured in the osteogenic medium for 21 days. Alizarin Red-positive calcium nodules were observed (Figure 1A), correlated with the upregulation of osteogenic markers Runx2 and OCN (Figure 1B). On the other hand, cells were cultured in the adipogenic medium for 14 days, and Oil Red O-positive lipid droplet was observed (Figure 1C). These above results revealed that hASCs had the characteristics of MSCs. To explore the expression of PC2 in hASCs during osteogenic differentiation, PC2 expression was detected by RT-PCR and immunofluorescence after cultured in osteogenic medium for 1-, 3-, or 5-day. The results showed that the expression of PC2 increased on the 3- and 5-day after osteogenic induction, indicating that the osteogenic differentiation was closely related to the expression of PC2 (Figures 1B,D).

### Effect of mechanical tension on proliferation of human adipose-derived stem cells

**FIGURE 2:**
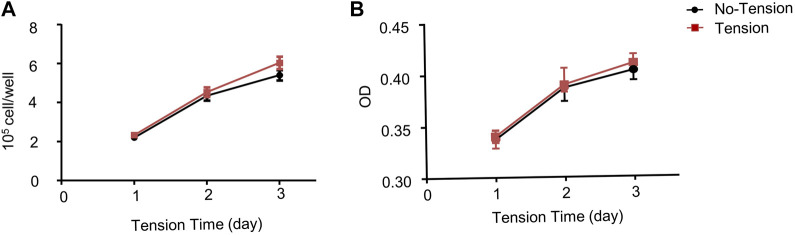
Effect of mechanical tension on proliferation of hASCs. **(A)** hASCs were cultured in growth medium for 1-, 3-, or 5-day with or without tension. The number of surviving cells was calculated **(B)** Proliferation ability of hASCSs was measured by Cell Counting Kit-8 (CCK8) assays. Values are the mean ± SEM. *p < 0.05, **p < 0.01. Experiments = 3.

To evaluate whether mechanical tension affected proliferative ability of hASCs, cell counting experiment and CCK8 analysis were performed. The cell counting experiment results showed that there was no difference in cell proliferation index between tension and no-tension groups at each time point (Figure 2A). The CCK8 analysis results also revealed that the proliferation of hASCs hardly increased in tension groups compared with no-tension groups (Figure 2B).

### Effect of mechanical tension on osteogenic differentiation of human adipose-derived stem cells

**FIGURE 3:**
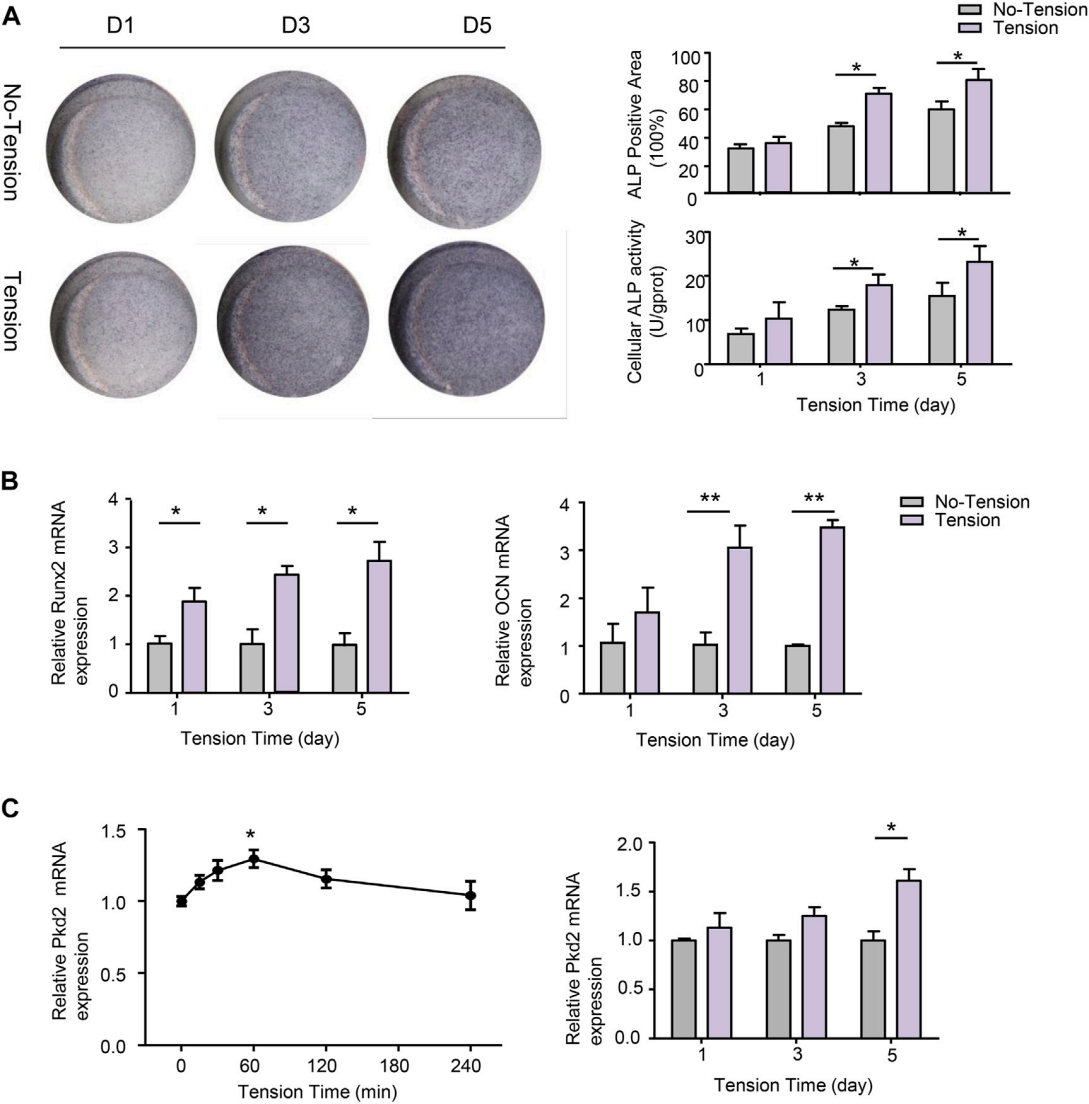
Effect of mechanical tension on osteogenic differentiation of hASCs. **(A)** hASCs were subjected to 1-, 3-, or 5-day’s mechanical tension. ALP staining was performed to visualize the osteogenic ability of hASCs.ALP^+^ areas were measured by ImageJ. Cellular ALP activity was detected by colorimetric assay **(B)** Osteogenic-specific makers Runx2 and OCN were detected at mRNA levels with real-time PCR after 1-,3-, or 5- day’s mechanical tension. GAPDH was used for normalization **(C)** After 0, 15, 30, 60, 120, or 240-min’s mechanical tension and 1-,3-, or 5- day’s mechanical tension, the mRNA level of PKD2 was examined by real-time PCR. GAPDH was used for normalization. Values are the mean ± SEM. *p < 0.05, **p < 0.01. Experiments = 3.

To evaluate whether mechanical tension influenced osteogenic differentiation of hASCs, ALP staining and ALP activity were performed after 1-, 3-, or 5-day’s mechanical tension. ALP staining showed that osteogenic differentiation of hASCs was enhanced by mechanical tension, which was significantly different from no-tension group. ALP activity was also gradually increasing and reached the peak on 5-day’s mechanical tension (Figure 3A). The certain osteogenic related markers including Runx2 and OCN were further investigated. RT-PCR results confirmed that mRNA levels of Runx2 and OCN were both upregulated after 3- and 5-day’s mechanical tension (Figure 3B). PC2 has been reported to be required for the anabolic response of chondrocytes to mechanical tension (Thompson et al., 2021). Thus, this study investigated whether PC2 is also required for the response of hASCs to short-term and long-term mechanical tension. In the short time, the mRNA expression of PC2 increased gradually and then declined, with the highest expression at 60-min’s mechanical tension. In the long time, PC2 expression also increased after 5-day’s mechanical tension (Figure 3C).

### Effect of Polycystin-2 knockdown on primary cilia formation of human adipose-derived stem cells

**FIGURE 4:**
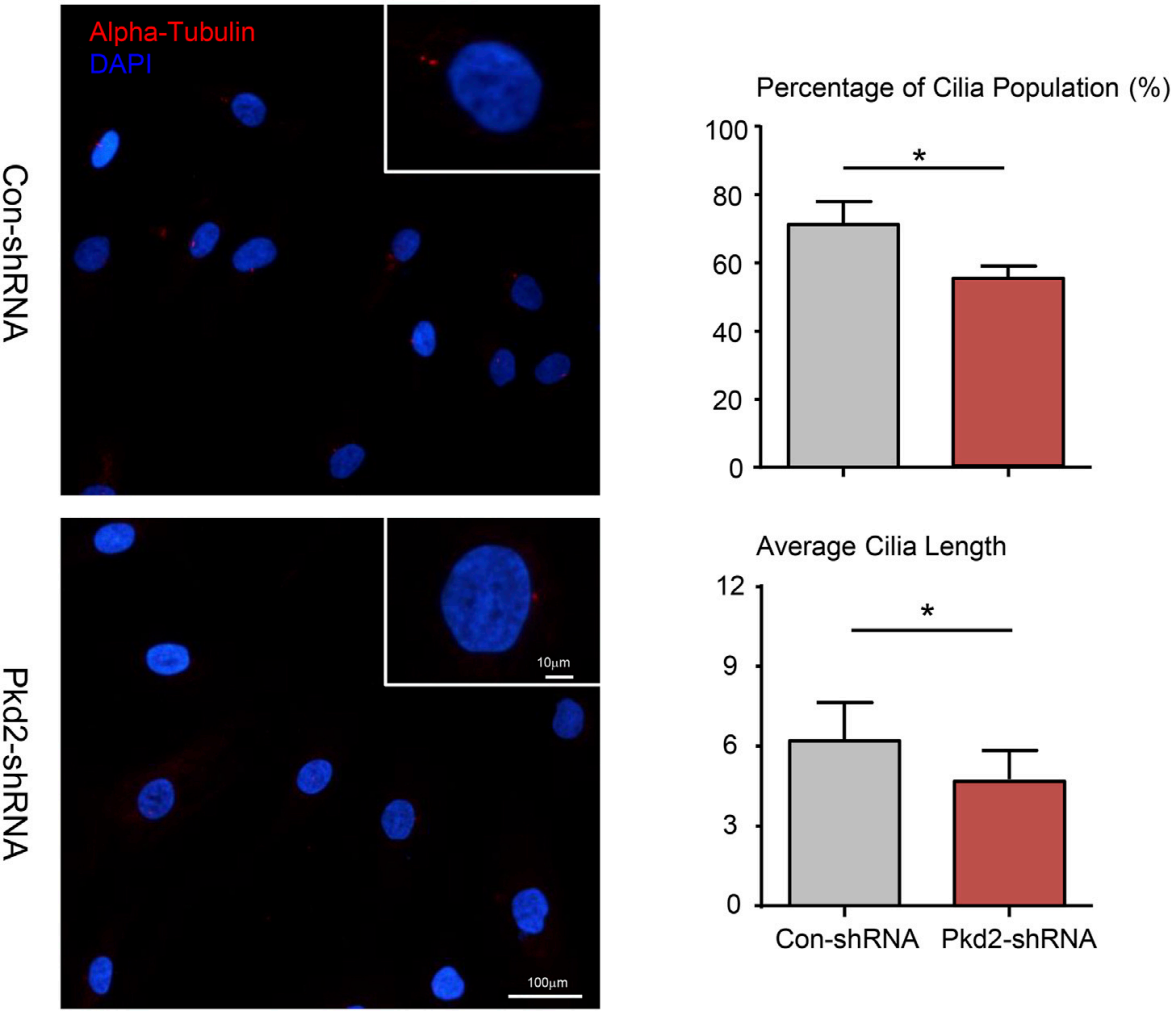
Effect of PC2 knockdown on primary cilia formation of hASC. After treated with PKD2 shRNA, primary cilia (red) subcellular localization in hASCs was observed by immunocytofluorescent. DAPI was used to highlight nuclei (blue). Scale bar: 10 µm. The cilia population frequency and the average cilia length were measured in hASCs. Values are the mean ± SEM. *p < 0.05, **p < 0.01. Experiments = 3.

Several studies suggested that PC2 is required for mechanotransduction of primary cilium (Nauli et al., 2003; Thompson et al., 2021). Therefore, we examined the effect of PKD2 shRNA on ciliary formation of hASC according to previous studies (Gardner et al., 2011). The results showed that PC2 knockout significantly reduced the cilia population frequency. In addition, the average cilia length was decrease in Pkd2-shRNA cells compared with con-shRNA cells (Figure 4).

### Polycystin-2 mediated mechanical tension-induced osteogenic differentiation

**FIGURE 5:**
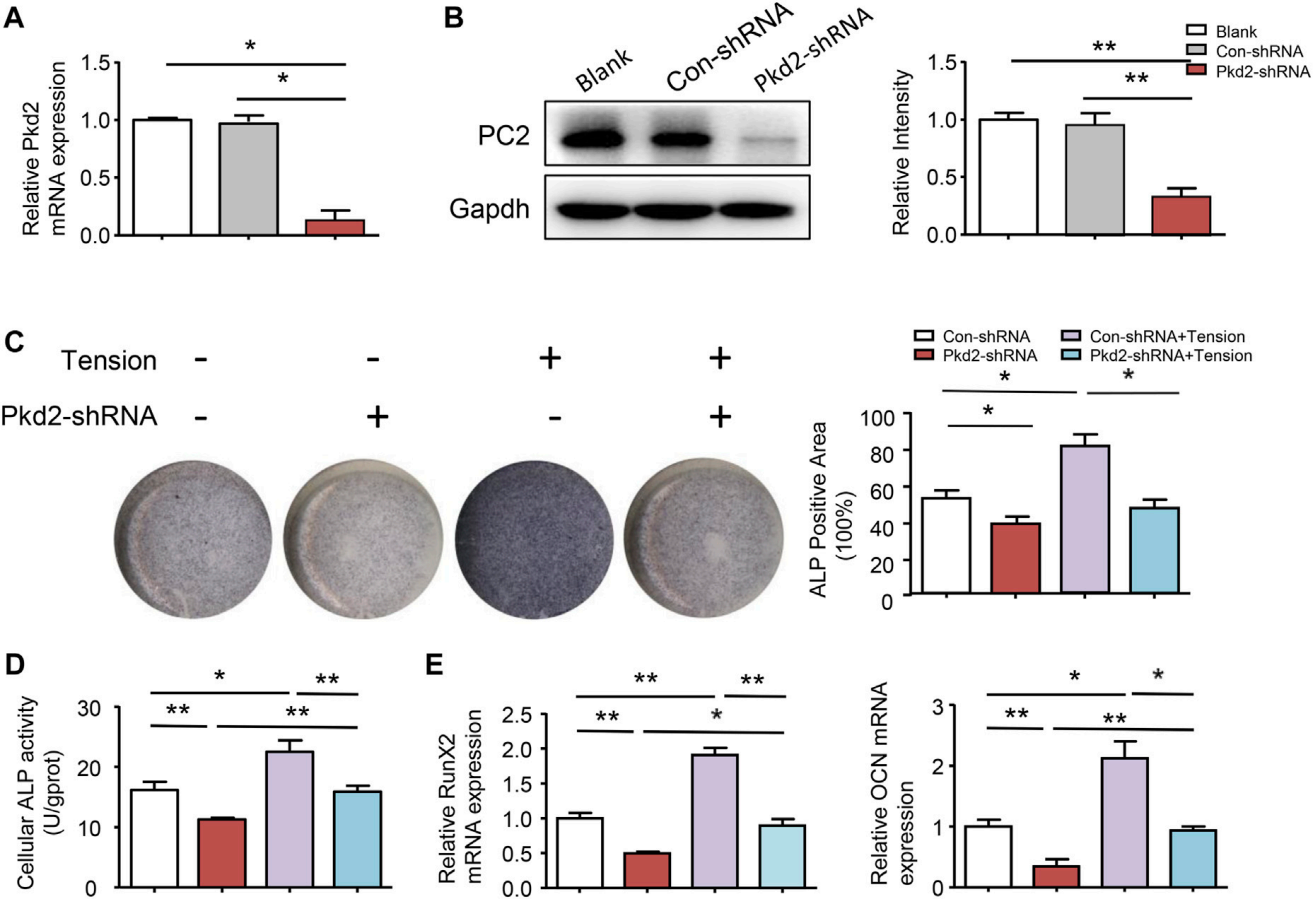
PC2 mediated mechanical tension-induced osteogenic differentiation. **(A) (B)** After treated with PKD2 shRNA, efficiency of lentivirus-shRNA interference was detected by real-time RT-PCR and western blot. **(C)** After hASCs were treated with Pkd2-shRNA and 5-day’s mechanical tension, ALP staining was performed and ALP^+^ areas were measured **(D)** ALP activity was detected by colorimetric assay **(E)** After 5-day’s mechanical tension, mRNA expression levels of Runx2 and OCN in hASCs transfected with Con-shRNA or PKD2-shRNA were examined by real-time PCR. GAPDH was used for normalization. Values are the mean ± SEM. *p < 0.05, **p < 0.01. Experiments = 3.

To further confirm the above results, PC2 was silenced in hASCs. Compared with control shRNA cells, PKD2 mRNA level decreased by 82% and PC2 protein level reduced 74% in PKD2 shRNA cells (Figures 5A,B). ALP staining showed that 5-days’ mechanical tension induced osteogenic differentiation of hASCs, which was suppressed by silencing PKD2 gene (Figure 5C). Additionally, ALP activity indicated the same tendency with ALP staining (Figure 5D). Compared with control shRNA cells, the mRNA expression of Runx2 and OCN was increased after 5-days’ mechanical tension, which was suppressed by silencing PKD2 gene (Figure 5E).

### Polycystin-2 mediated mechanical tension-induced transcriptional co-activator with PDZ-binding motif nuclear translocation and osteogenic differentiation

**FIGURE 6:**
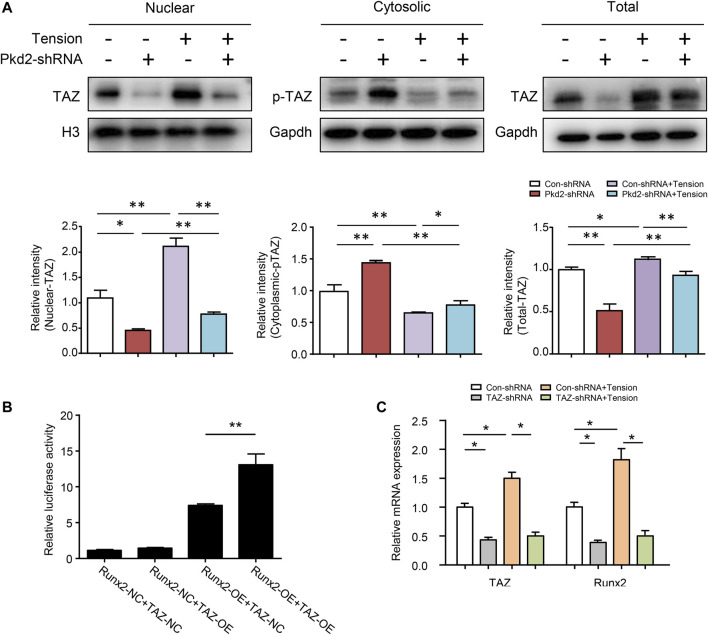
PC2 mediated mechanical tension-induced TAZ nulear translocation and osteogenic differentiation. **(A)** After 5-day’s mechanical tension, the levels of nuclear TAZ, cytosolic p-TAZ and total TAZ were examined by western blot in hASCs treated with Con-shRNA or Pkd2-shRNA. Histone H3 was used as nuclear loading control. GAPDH was used as cytosolic and total protein loading control **(B)** Luciferase reporter assays in 293T cells illustrated that TAZ could bind with Runx2 at gene level **(C)** After 5-day’s mechanical tension, the mRNA levels of TAZ and Runx2 were detected in hASCs treated with Con-shRNA or TAZ-shRNA. GAPDH was used for normalization. Values are the mean ± SEM. *p < 0.05, **p < 0.01. Experiments = 3.

Previous studies reported that PC2 could modulate the cellular sublocation of TAZ (Xiao et al., 2014). Our results revealed that the protein expression of total TAZ and nuclear TAZ was decreased upon PKD2 shRNA was used. In contrast, cytoplasmic phosphorylated TAZ was increased by PKD2 shRNA. TAZ activity has been extensively characterized in regulating bone growth and development, and it can modify behaviors of cells exposed to mechanical stimuli (Zhu et al., 2018; Li et al., 2020). Since the nuclear localization of TAZ is mediated by its dephosphorylation, the levels of TAZ and phosphorylated TAZ were detected respectively in nucleus and cytoplasm after 5-day’s mechanical tension. The results showed that mechanical tension significantly increased nuclear TAZ expression and decreased cytosolic phosphorylated TAZ level. Moreover, both total TAZ and nuclear TAZ up-regulated by mechanical tension were suppressed by silencing PKD2 gene (Figure 6A). It is reported that transcriptional co-activators TAZ could interact with Runx2, which can regulate osteogenesis of MSCs(Hong and Yaffe, 2006; Li et al., 2020). To further identify the combination in hASCs, natural control and overexpression double fluorescent reporters of Runx2 promoter and transcription factor TAZ were constructed. Then the binding capacity of promoter and transcription factor was detected in 293T cells. The result indicated that promoter Runx2 and transcription factor TAZ had direct combination sites (Figure 6B). To further understand the effect of TAZ on tension-induced Runx2 upregulation, hASCs were transfected with lentivirus to knock-down TAZ. The mRNA level of TAZ reduced and mechanical tension-induced upregulation of Runx2 was subsequently suppressed (Figure 6C). Thus, the above results suggested that mechanical stimuli could induce TAZ nuclear translocation, and PC2 played a key role in this regulatory process. The nuclear TAZ had direct combination with downstream Runx2 which subsequently increased tension-induced osteogenesis.

## Discussion

Currently, both regenerative medicine and tissue engineering are exploiting the therapeutic potentials of stem cells for bone repair and regeneration (Ntege et al., 2020). More and more evidences show that hASCs, as a stem cell population with multidirectional differentiation potential, can promote tissue repair through direct or indirect mechanisms (El-Sabbagh, 2017; Bacakova et al., 2018). Mechanical stimuli is one of the methods to promote osteogenesis of stem cells (Marrelli et al., 2018; Fang et al., 2019). However, there are few studies conducted to explore the osteogenic performance and underlying mechanisms of hASCs under mechanical tension. Understanding the underlying physiological mechanisms has important guiding significance for the treatment of musculoskeletal diseases and injuries. Here, our results revealed that mechanical tension significantly promoted osteoblastic differentiation and PC2 was critically required for mechanical tension-induced osteogenesis differentiation of hASCs by activating TAZ.

Exogenous mechanical stimuli could induce the physiological responsiveness of various cells and play an important role in regulating the structure and function of cells (Guo et al., 2020; Li et al., 2020). Flexcell^®^ FX- 5000™ Tension System is a computer-regulated bioreactor, which applies cyclic or static strain to cells on flexible-bottomed cultures plates through the use of vacuum pressure (Wu et al., 2018). Since hASCs, which cultured on Silicone Rubber Bioflex^®^ Culture Plates, has grown too densely on the fifth day, we only conducted experiments within 5 days. Moreover, it has been proved that application of 10% cyclic tensile strain has an enhanced osteogenic phenotype (Charoenpanich et al., 2011; Fang et al., 2019). We also applied 10% mechanical tension to hASCs and found that mechanical tension could promote the osteogenic differentiation of hASCs, suggesting that mechanical tension indeed could promote the osteogenic differentiation ability of hASCs. However, the proliferation of hASCs was not affected by mechanical tension. This was in agreement with the findings of Li et al. (Li et al., 2020) who found that mechanical tension could not promote the proliferation of cranial suture mesenchymal stem cells, but it was contrary to the research of Choi et al. (Choi et al., 2007). Their results showed that cyclic strain applied to hASCs enhanced their proliferation in the early stage. The possible reason for these differences may be related to the mechanical parameters and the time length for mechanical tension.

PC2 acts as a nonselective cation channel, which can trigger a variety of biological processes including chemical activity and signaling pathways (Gees et al., 2010). Evidence from literature has depicted that PC2 is required for anabolic gene expression in response to cyclic tensile strain (Thompson et al., 2021). Therefore, we explored the expression of PC2 in hASCs after osteogenic induction, and found that osteogenically stimulated hASCs expressed more PC2. Additionally, our study found that PC2 increased gradually and then declined under the short-term mechanical tension and also increased after 5-day’s long-term mechanical tension. Silencing PKD2 gene of hASCs exhibited an inhibited response to mechanical stimuli, resulting in an abolition of strain-induced increase of osteogenic differention. These results demonstrated that PC2 plays an important role in the response of bone to mechanical tension.

Primary cilia has also been considered as an important mechanical signal organelle (Basten and Giles, 2013; Bodle et al., 2013), which can detect exogenous stimuli and convert signals into a variety of cell biochemical activities, such as proliferation, differentiation and migration (Nguyen and Jacobs, 2013; Yan et al., 2015). Bodle et al. (Bodle et al., 2019) determined the role of cilia in the mechanical signal transduction of ASCs, finding that cilia dynamically modulates mechanical stimuli-induced hASCs diferentiation, and the loss of primary cilia prevents the upregulation of osteogenesis under mechanical stimuli. Meanwhile, PC2 can interact with polycystin-1 (PC1) to form a complex that co-localizes to the primary cilia in many cells, thereby creating a “sensor” that regulates bone mass (Xiao et al., 2011; Winokurow and Schumacher, 2019). Therefore, we explored the changes of the cilia after silencing PKD2 gene and verified that down-regulating PC2 caused a reduction in the cilia population frequency and cilia length of hASCs. The results were corresponded with Thompson et al. (Thompson et al., 2021), who found that robust cilia disassembly was observed in Pkd2-siRNA treated chondrocyte. Thus, silence of PKD2 gene could partially destroy primary cilia formation of hASCs in our study.

TAZ knockout in zebrafish contributes to the failure of bone formation (Hong et al., 2005). TAZ activity is regulated by a phosphorylation event that can modify behaviors of cells exposed to mechanical stimuli (Byun et al., 2012; Li et al., 2020). Our study revealed that mechanical tension could promote nuclear translocation of TAZ. Previous evidences have verified that TAZ cooperates with Runx2 to promote osteogenesis of mesenchyme stem cells (Hong et al., 2005; Li et al., 2020). We did observe a direct combination of TAZ and Runx2 in hASCs, and this combination was involved in the increase of mechanical tension-induced osteogenesis. It is reported that not only PC2, but also TAZ play an important role in determining the differentiation of mesenchyme stem cells (Dupont et al., 2011; Thompson et al., 2021). PKD2 mutations caused alveolar bone loss and anomalies in cranial development (Khonsari et al., 2013). The bone phenotypes associated with perturbations of both PC2 and TAZ expression indicate that these proteins have the possibility to participate together in a common signaling pathway. Indeed, our discovery is that silence of PKD2 gene would block TAZ dephosphorylation and nuclear translocation when mechanical tension was applied, which is in accordance with Xiao et al. (Xiao et al., 2014). We believed that TAZ played a key role in determining the fate of hASCs under mechanical tension. PC2 appears to be an important part of which may directly adjust the mechanical signal conduction during the mechanical tension-induced osteogenic differentiation of hASCs. However, the deeper molecular mechanism in this study has not yet been clarified and needs to be further explored. It is possible to further clarify that the mechanism function of PC2 is likely to provide new cilia’s targets for drug discovery and promotion of bone.

## Data Availability

The original contributions presented in the study are included in the article/supplementary material, further inquiries can be directed to the corresponding authors.
